# Supplementary material for: Modulation of initial leftward bias in visual search by parietal tDCS
Source: PLoS One. 2024 Dec 31;19(12):e0315715. doi: 10.1371/journal.pone.0315715 (PMC11687727; doi:10.1371/journal.pone.0315715)
Supplement: S1 Appendix — (DOCX) [file pone.0315715.s001.docx]

**Modulation of initial leftward bias in visual search by parietal tDCS:**

**S1 Appendix.** Detailed statistics of the LMM on the CoC in the cancellation tests.

| **Fixed-effect** | ***Χ²*** | **df** | **p-value** |
| --- | --- | --- | --- |
| Test | *8.54* | *2* | *<.014** |
| Hemisphere | *0.74* | *1* | *.389* |
| tDCS | *0.07* | *1* | *.792* |
| Test x Hemisphere | *0.99* | *2* | *.609* |
| tDCS x Hemisphere | *0.05* | *1* | *.828* |
| Test x tDCS | *0.06* | *2* | *.971* |
| Test x tDCS x Hemisphere | *0.12* | *2* | *.941* |

*** *p*-values < .001; ** *p*-values < .01; * *p*-values < 0.05
