# Supplementary material for: Modulation of initial leftward bias in visual search by parietal tDCS
Source: PLoS One. 2024 Dec 31;19(12):e0315715. doi: 10.1371/journal.pone.0315715 (PMC11687727; doi:10.1371/journal.pone.0315715)
Supplement: S2 Appendix — (DOCX) [file pone.0315715.s002.docx]

**Modulation of initial leftward bias in visual search by parietal tDCS:**

**S2 Appendix.** Detailed statistics of the Gamma GLMM on the percentage of crossed targets in the cancellation tests.

| **Fixed-effect** | ***Χ²*** | **df** | **p-value** |
| --- | --- | --- | --- |
| Test | *1045.70* | *2* | *< .001**** |
| Hemisphere | *0.13* | *1* | *.715* |
| tDCS | *0.48* | *1* | *.489* |
| Test x Hemisphere | *1.79* | *2* | *.407* |
| tDCS x Hemisphere | *4.81* | *1* | *.028** |
| Test x tDCS | *0.09* | *2* | *.957* |
| Test x tDCS x Hemisphere | *3.61* | *2* | *.164* |

*** *p*-values < .001; ** *p*-values < .01; * *p*-values < 0.05
