# Supplementary material for: Modulation of initial leftward bias in visual search by parietal tDCS
Source: PLoS One. 2024 Dec 31;19(12):e0315715. doi: 10.1371/journal.pone.0315715 (PMC11687727; doi:10.1371/journal.pone.0315715)
Supplement: S3 Appendix — (DOCX) [file pone.0315715.s003.docx]

**Modulation of initial leftward bias in visual search by parietal tDCS:**

**S3 Appendix.** Detailed statistics of the binomial GLMM on the accuracy at the Cloud task.

| **Fixed-effect** | ***Χ²*** | **df** | **p-value** |
| --- | --- | --- | --- |
| Similarity | *177.79* | *2* | *< .001**** |
| Hemisphere | *0.93* | *1* | *.333* |
| tDCS | *0.01* | *1* | *.916* |
| Similarity x Hemisphere | *2.40* | *2* | *.301* |
| Similarity x tDCS | *1.08* | *2* | *.583* |
| Hemisphere x tDCS | *0.36* | *1* | *.547* |
| Similarity x tDCS x Hemisphere | *1.32* | *2* | *.516* |

*** *p*-values < .001; ** *p*-values < .01; * *p*-values < 0.05
