# Supplementary material for: Modulation of initial leftward bias in visual search by parietal tDCS
Source: PLoS One. 2024 Dec 31;19(12):e0315715. doi: 10.1371/journal.pone.0315715 (PMC11687727; doi:10.1371/journal.pone.0315715)
Supplement: Attachment — Submitted filename: GEERS_Response_to_Reviewers.docx [file pone.0315715.s004.docx]

**Reviewer #1**

We thank both reviewers for their insightful comments that draw our attention on the importance of the starting point. Beyond its influence on the visual search strategy, the starting point has proved to be a relevant dependent variable we had initially overlooked. In response to the reviewers’ questions, we extracted the average x-coordinates of the first mark and then had the idea to further investigated the effect of tDCS on the position of the first cancelled target, which is known to be a good indicator of neglect in patients. The results showed that anodal stimulation of the parietal cortex resulted in a significant shift of the position of the first cancelled target by nearly 100 pixels in the contralateral direction. This finding suggest that participants started from a different position as a result of a hemispheric imbalance induced by tDCS. This finding has been incorporated in the revised manuscript and, given its scientific and clinical significance, it has also been reflected in the title. Note that we have kept track of all the reviewers’ remarks in the revised manuscript where this new finding is put into perspective with other aspects of the results.

*In two sessions, either tDCS or sham stimulation was applied in healthy participants, on the left hemisphere (n = 23) or right hemisphere (n = 23). Effect of the stimulation on visual search and visual imagery was evaluated, specifically focusing on the visuospatial asymmetry in performance. Anodal tDCS was applied over the parietal cortex, increasing the spontaneous firing rate, assumed to improve visual detection in the contralateral hemifield. The study is relevant to inform treatments of neglect, and conducted properly. I do have some suggestions that the authors may want to take into account.*

*1. The authors mention that the cancellation tasks are sensitive to pseudoneglect, especially since a time limit was used. Although I agree that there probably is an effect of pseudoneglect, I expect that most Western participants use a strategy starting at the left side, and working their way towards the right side, in line with the culturally defined habitual reading direction (left-to-right). I am aware that there are many studies showing that a leftward bias exists even when there is no reason to believe that reading direction would affect this (e.g., if there is no search or scanning involved), and that there are studies showing that people who are used to a right-to-left reading direction still show a leftward bias, but specifically for the cancellation task I can imagine that reading direction and strategy will have such a large influence that it might overrule the more subtle leftward bias. Especially since at some point there simply is no time left, and participants cannot complete their search towards the right side of the template, missing the targets there.*

*1a. Can the authors comment on this, and include some literature on effects of reading direction on visual search?*

The reviewer is right to highlight the potential influence of reading habits on our findings. Several studies have shown that most adults cancel the first target on the left side (1–4). This leftward bias was not present in young children (around 3 years old) but increased with age, suggesting that it is learned through reading habits (5). Consistent with this idea, the starting point for target cancellation is influenced by reading direction. Left-to-right readers (e.g., Italians) were found to start more often on the left side, whereas right-to-left readers (e.g., Israelis) showed no bias or a slight rightward bias. However, the initial rightward bias of right-to-left readers did not simply mirror the leftward bias of left-to-right readers, suggesting a moderating effect of neurobiological asymmetries that orient visuospatial attention to the left side of space according to Rinaldi and colleagues (6).

In our study, we also observed an initial leftward bias, with the average (± SD) x-coordinate of the first cancellation mark being 121 (± 323) pixels on the left of the center of the template. We cannot exclude that part of this bias arose from the reading habits of our participants, who were left-to-right readers. However, we found that this left initial bias was modulated by tDCS, so that it was enhanced (more lateral) during anodal stimulation of the right parietal cortex and reduced (more central) during anodal stimulation of the left parietal cortex. Notably, tDCS shifted the starting position by nearly 100 pixels, compared to sham condition, suggesting that participants selected a more lateral or central target as a result of the hemispheric imbalance caused by tDCS. The two-way interaction between condition and hemisphere, *χ*²(1) = 6.75, *p* = .009, was integrated in a three-way interaction with test χ²(2) = 8.88, *p =* .012, indicating that this effect was observed in the Mesulam test and, to a lesser extent, in the Star test, but not in the Ota test (see detailed results on pages 13-15). Hence, our results suggest that at least part of the left initial bias is due to neurobiological asymmetries that can be modulated with tDCS.

We have added literature about the influence of reading habits on the initial bias in visual search in the Introduction section (page 5), the described analysis in the Methods (pages 10-11) and Results (pages 13-15) sections and discussed them together in the Discussion section (page 21).

*1b. In the current study, did participants typically reach the right side of the template?*

Yes, participants crossed the midline in most trials, regardless of whether they started from the left or right side of the template. This occurred, on average, 11.7 ± 6.8 seconds after the trial onset, i.e., before the middle of the trial whose total duration was 30 seconds, indicating that the lower proportion of targets crossed on the right side cannot be attributed to a lack of time. This pattern is also visible when computing a center of cancellation (CoC) for each 5-second time bin. As shown in the figure below, regardless of whether the first target cancelled was on the right or left, the CoC progressively shifted towards the opposite direction and changed sign before the middle of the trial (between bins 3 and 4). This strategy left enough time to cross as many targets as on the other side during the remaining time. However, it is noticeable that the CoC is less polarized during the second half of the trial (absolute value < 0.4) than the first half (absolute value > 0.5), indicating that participants did not go as far on the side opposite to the starting side. The most reasonable explanation is that participants did not progress linearly towards the other side but still crossed some targets on the starting side due to loops and revisits of already explored locations, as visible in the individual data that have been added in the Open Science Framework (https://osf.io/4dbs5/). This is now discussed on page 21 of the manuscript.

**Figure 1.** The average center of cancellation (CoC) for each 5-second time bin as a function of the starting side (left in red and right in blue). Positive and negative values represent, respectively, a rightward and leftward CoC. The grey line represent the CoC averaged over starting positions.

*1c. Was there a relation between the CoC and the starting point (left or right)? This will provide insight into the effect of the starting point on the CoC.*

Yes, we found a significant positive relationship between the CoC and x-coordinate of the first cancellation mark in the sham condition. Specifically, a more leftward starting position resulted in a more leftward CoC, *χ*²(1) = 27.97, *p* < .001. This relationship persisted in the active tDCS condition, *χ*²(1) = 53.65, *p* < .001, despite the fact that tDCS modulated the initial leftward bias and not the CoC. Thus, the processes that sustain the visual search are partially distinct from those that guide the selection of the first cancelled target as it is possible to modulate the starting position independently of the CoC. Importantly, as mentioned previously, this result is not due to a lack of time to visit both sides of the template. This is now discussed on page 21 of the manuscript.

*1d. Furthermore, the CoC values are very high (values below -0.25 are not uncommon) which further stresses that no subtle left-right difference is reflected, but more likely a much larger effect of strategy.*

High CoC values have indeed been observed in some individuals and we acknowledge that the CoC might have been enhanced by the strategy of the participants. An intricate question now discussed in the paper is to which extent the adopted strategy reflects neurobiological (i.e. a left-right hemisphere imbalance) or cultural (i.e. reading habits) factors (page 21). As pseudoneglect is generally associated with a left-right hemisphere imbalance (7), we decided to replace the term “pseudoneglect” by “leftward bias” to not bias the conclusions to one or the other account.

**Minor points**

*- Please add the number of participants in the abstract.*

The number of participants has been added in the abstract.

*- Is there information on the test-retest reliability in healthy controls for the tests that are used? It would be useful to include this information.*

Cancellation tests typically exhibit high test-retest reliability regarding the number of targets crossed (8,9). In the current study, the experimental design was not ideally suited for assessing test-retest reliability due to the use of different (i.e., flipped) versions of the tests across repetitions. Despite this limitation, we calculated Pearson correlation coefficients to evaluate the consistency of each dependent variable between the normal (first) and flipped (second) versions of each test during the sham session. For the x-coordinate of the first cancelled target, we observed a significant positive correlation for each test (Mesulam: *r* = 0.38, *p* = .007; Ota: *r* = .72, *p* < .001; Star: *r* = .72, *p* < .001). Regarding the CoC, a significant correlation was found only for the Star test, *r* = .37, *p* = .011, whereas no significant correlations were observed for the Mesulam, *r* = .01, *p* = .948, and Ota, *r* = -.18, *p* = 0.211, tests. Finally, for the percentage of crossed targets, positive correlations were evident for the Mesulam, *r* = 0.63, p <.001, Ota, *r* = 0.70, p < .001, and Star, *r* = 0.60, *p* < .001, tests. These results align with previous findings, supporting the good test-retest reliability of cancellation tests, except for the CoC in the Mesulam and Ota tests. These results have been added to the manuscript (page 18) but should however be interpret with caution, as the reliability was assessed using flipped versions of the tests.

*- Report the statistical results, including exact p-values and effect sizes, for all the post-hoc tests.*

This has been addressed in the revised manuscript.

*- Why was only the Mesulam cancellation test correlated with the LQ of the Cloud task, and not the other cancellation tests?*

The correlation is less reliable for the Star and Ota cancellation tasks due to a ceiling effect in the number of crossed targets, reducing the sensitivity of these tasks (i.e. less inter-individual variability). However, for the sake of completeness, we now report the correlation for these two tasks as well, which were also not significant (page 19).

*- The figures are informative, good that the individual data points are presented. However, it would be more complete to include figures for all the other outcome variables. In addition, it would be informative to also include the group means and error bars in the graphs.*

We now provide figures for all the outcome variables, including initial starting position, with the mean group performance and related standard error superimposed (in black) on the individual data (in light grey).

*- The figure captions can include more information, such as the number of participants per group, and what the horizontal line at “0” indicates.*

This information has been added in the revised manuscript. Thank you for drawing our attention on the missing information.

**Reviewer #2**

*Geers and colleagues investigated the effect of anodal tDCS applied over left or right parietal cortex on visual search and imagery abilities in a sample of healthy participants. The authors reported no effect of tDCS on neither visual search, visual imagery nor visuospatial bias, thus concluding that anodal tDCS of the parietal cortex was not sufficient to alter the exploration of visual and mental images and to counteract spatial bias in visual search.*

*I have some major issues mainly regarding the methodology adopted to investigate pseudoneglect, the sample size calculation and the statistical analysis performed that prevent me to endorse the paper for publication in its present form. Below my main concerns.*

*1. Even if cancellation tests represent a clinical gold standard for assessing neglect, the way it is scored in the present study does not allow to fully capturing the potential modulatory effect on visuospatial asymmetries. Indeed, with a 30 sec time limit, it is possible that right side omissions will always be greater to left side omissions because of the cultural left-to-right reading and writing habits. Indeed, native language direction modulates spatial bias having an effect on the side of the omissions (e.g. Rinaldi et al., 2014). Furthermore, in visual search and cancellation tasks a preference to direct the first eye movement in the upper left quadrant has been reported (Behrmann, Watt, Black, & Barton, 1997; Weintraub & Mesulam, 1988). Accordingly, Table 1 clearly indicates a greater number of crossed targets in the left visual field in all conditions. In my opinion, the methods implemented in the present study has the merit of showing the impact of tDCS on visual search (i.e., cancellation of targets among distractors) but they do not represent an adequate model to measure its effect on pseudoneglect.*

We would like to thank the reviewer for drawing our attention on the importance of the starting point, which allowed us to maximize the insights from our data. Several studies have indeed reported that most adults cancel the first target on the left side of the template (1–4). This leftward bias was absent in young children (around 3 years old) but increased with age, suggesting that it develops through reading habits (5). Consistent with this idea, the starting point for target cancellation is influenced by reading direction. Left-to-right readers (e.g., Italians) were found to start more often on the left side, whereas right-to-left readers (e.g., Israelis) showed no bias or a slight rightward bias. However, the initial rightward bias of right-to-left readers did not simply mirror the leftward bias of left-to-right readers, suggesting a moderating effect of neurobiological asymmetries that orient visuospatial attention to the left side of space according to Rinaldi and colleagues (6).

To determine whether such a bias in the starting position was present in our study, we extracted the average x-coordinates of the first marks, which were, on average, 121 pixels to the left of the center of the template. We then had the idea to further investigated the effect of tDCS on the position of the first cancelled target, which is known to be a good indicator of neglect in patients. The results showed that anodal stimulation of the parietal cortex caused a significant shift of nearly 100 pixels in the contralateral direction for the first cancelled target. This finding suggest that participants started from a different position due to the interhemispheric imbalance induced by tDCS. The two-way interaction between condition and hemisphere, *χ*²(1) = 6.75, *p* = .009, was integrated in a three-way interaction with test χ²(2) = 8.88, *p =* .012, indicating that this effect was observed in the Mesulam test and, to a lesser extent, in the Star test, but not in the Ota test (see detailed results on pages 13-15). We cannot exclude the possibility that part of this bias arises from our participants being left-to-right readers, as it is challenging to disentangle the effect of reading habits from neurobiological asymmetries. However, the observed effect of tDCS suggests that this initial leftward bias cannot be entirely attributed to reading habits and that interhemispheric balance, which can be modulated through parietal stimulation, also plays a role.

Furthermore, the leftward bias in the spatial distribution of cancelled targets (or CoC) is unlikely to be explained by time constraints limiting progression to the right following the initial leftward bias. The total duration of one trial was fixed at 30 seconds, and participants crossed the midline, on average, 11.7 ± 6.8 seconds after the trial onset, allowing sufficient time to cross as many targets on the opposite side as on the starting side. A close examination of individual traces (see individual cancellation sheets made available at https://osf.io/4dbs5/) revealed more complex strategies than simply vertically or horizontally scanning the template. These strategies include looping or revisiting already explored locations, which might also explain why the CoC is biased towards the starting side.

In summary, the leftward bias highlighted by the reviewer has been reported in the revised manuscript, with the related literature documenting this bias. We acknowledge that this bias does not only reflect visuo-spatial asymmetries but potentially also reading habits. We have carefully described the possible sources of this leftward bias to prevent misinterpretations (page 5). We have further elaborated on the finding that this leftward bias can be modulated by tDCS as participants select the first target (page 20). Consequently, we have also decided to replace the term "pseudoneglect," which is generally associated with these asymmetries (7), with "leftward bias," which directly refers to what we observe in the data. Finally, we have discussed the possible influence of time limits on an leftward CoC, remaining unaffected by tDCS (page 21).

2. It is not clear to me how effect size for sample size calculation has been obtained. Indeed, authors provided no information on the presumed effect size. Based on which findings the authors considered a medium effect size (f=0.25)? Considering the concerns of reliability of tDCS effects, mainly due to reduced replicability and to interindividual variability in responses, sample size estimation is crucial, with underpowered studies being a potential reason for the reported inconsistences.

No prior information was available about effect size as this is the first study investigating the effects of parietal tDCS on visual search and mental imagery in healthy controls. The sample size calculation was estimated a priori based on a medium effect size because we looked for a non-negligible, useful, and theoretically meaningful effect. In particular, we were interested in effects sufficiently large to motivate the use of this protocol in brain-lesioned patients. This has been specified in the revised manuscript (page 7). A medium-size effect seems reasonable with regard to the results of previous studies assessing the effect of parietal tDCS on other visuospatial tasks, and sample size in our study is not on the low side compared to what is usually considered as a convenient sample in the literature (10–12). Finally, although this has not the same value as an a priori sample size calculation, the supplementary analyses on the x-coordinate of the first cancelled target indicate that our study was sufficiently powered to detect all two-way interactions, as well as the three-way interaction, between condition, hemisphere and test. Note the study power was further increased since we used gamma-corrected generalized linear mixed models to take into account the reviewer’s remark about the data distribution (see below). To sum up, we agree with the reviewer that replicability of tDCS effects is matter of concern but, in the present study, the sample size calculation seems fully justified given the research objectives and the study was not underpowered given the analyses performed.

3. Authors wrote that statistical analysis was performed on the percentage of crossed targets but Table 1 and Supporting Information 1 report the mean number of crossed targets. Please clarify this point.

We thank the reviewer for drawing our attention on this error which has been corrected. The analyses were indeed performed on the percentage of crossed targets to take into account the different number of targets included in each visual search test. All tables and figures now report the percentage of crossed targets to fit the analyses.

4. Post-hoc comparisons to investigate the significant “test” x “tDCS” x “hemisphere” interaction have been performed by comparing the number of crossed targets (or percentage?) between anodal and sham tDCS, not considering the “hemisphere” factor. Did authors performed an ANOVA with the factors “tDCS” and “hemisphere”, separately for each cancellation task?

We decomposed the three-way interaction by comparing anodal and sham tDCS separately for each combination of hemisphere and test, resulting in six comparisons in total. Thus, pairwise comparisons were run on the percentage of crossed targets during anodal and sham tDCS. We apologize if this was not clear in our original phrasing.

In the revised manuscript, we re-analyzed the data using a (gamma-corrected) generalized linear mixed model (GLMM) in order to address the comment of the reviewer on the normality data distribution (see next comment). The GLMM comes with pairwise comparisons assessing differences between each combination of tdcs, hemisphere, and test. Decomposing the three-way interaction revealed by the GLMM by running a new model coding the two-way interaction separately for each test would not be statistically sound because the estimated means used for pairwise comparisons would deviate from those estimated by the omnibus model. Please note that, if ANOVAs had been reported in the revised version, the decomposition suggested by the reviewer would have corroborated the finding that the Ota test gave rise to an interaction between tdcs and hemisphere, *F*(1,46) = 5.01, *p* = .030, and not the two other tests (Fs<1).

5. No mention is made regarding data distribution. Did authors check for normality of data distribution before performing parametric statistics?

The distribution of the data deviated from the normal distribution regarding the x-coordinate of the first mark, (skewness ± SE: 0.52 ± 0.10), *z* = 5.14, *p* < .001, and overall accuracy (-0.47 ± 0.10), *z* = 4.58, *p* < .001, in the three cancellation tests. While an ANOVA is fairly robust to moderate departures from normality (13,14), we have now opted for a Gamma Generalized Linear Mixed Model (GLMM). This model specifically accommodates skewness and offers greater sensitivity (see comment 2).

6. In the discussion section, authors did not consider all the tDCS-related factors possibly affecting tDCS effects on behavioral performance (e.g. intensity, montage, online vs offline protocols, state-dependency, baseline capacity, etc.). Please consider adding these considerations in the discussion.

We made clear that the present results hold only for the specific protocol we used and we discussed the potential contribution of other protocols in reference to the most recent literature. Among the critical parameters currently scrutinized in the domain, bilateral or dual tDCS seems to be more efficient than anodal stimulation alone. Moreover, it has been argued that current strength may interact (15) with baseline performance, but this has been recently refuted based on a conceptual replication study (16). Finally, in the present study, anodal stimulation was delivered continuously during task performance but started 2 minutes prior to the first task. We did so to anticipate a possible delay in the depolarization of the neural tissue but admittedly this delay could be longer than expected and the observed effects in visual search could actually be enhanced with off-line protocols that temporally dissociate the stimulation and the testing, as in previous studies on visuospatial detection. All these considerations have been extensively discussed in the revised version of the manuscript (pages 24-25).

Minor issues:

*- No mention is made regarding handedness. Please provide this information and whether it has been assessed with Edinburgh Handedness questionnaire.*

Handedness was already mentioned in the ‘Participants’ section. We have now further indicated that this information has been self-reported by the participants (not with the Edinburgh Handedness Inventory; page 7).

*- How was randomization (i.e., left vs right hemisphere stimulation) performed?*

Four possible orders were defined: (1) left tDCS on day 1 and left sham on day 2, (2) left sham on day 1 and left tDCS on day 2, (3) right tDCS on day 1 and right sham on day 2, and (4) right sham on day 1 and right tDCS on day 2. These four orders were systematically counterbalanced across the 48 participants. This clarification has now been included in the manuscript (page 10).

- A table with CoC results is missing.

A table presenting all the results, including the CoC results, has been added in the manuscript (page 14).

- P-values reported in the manuscript are not consistent with those reported in the Supporting Information (e.g., p. 12 line 263: p=0.025; supporting information 2: p=0.041).

Thank you for noticing this typo, which has now been corrected.

- The authors applied an online tDCS protocol, with participants performing tasks within the 20 minutes of stimulation. How long after the start of the stimulation did the tasks begin? How long did the tasks last? Please clarify this point

The instructions were given before positioning the electrodes. Then the stimulation started, with a 30 sec ramping up current, followed by 1 min of practice trials that were not analyzed. The Albert cancellation task was used as practice. After a fixed delay of 2 minutes from stimulation onset, participants started the experimental tasks whose completion fitted the 20 min of tDCS. This has been clarified (page 10).

**References**

1.	Manly T, Dove A, Blows S, George M, Noonan MP, Teasdale TW, et al. Assessment of unilateral spatial neglect: Scoring star cancellation performance from video recordings—method, reliability, benefits, and normative data. Neuropsychology. 2009;23(4):519.

2.	Rousseaux M, Beis JM, Pradat-Diehl P, Martin Y, Bartolomeo P, Bernati T, et al. Présentation d’une batterie de dépistage de la négligence spatiale. Rev Neurol. 2001;157:1385‑400.

3.	Nurmi L, Kettunen J, Laihosalo M, Ruuskanen EI, Koivisto AM, Jehkonen M. Right hemisphere infarct patients and healthy controls: Evaluation of starting points in cancellation tasks. Journal of the International Neuropsychological Society. 2010;16(5):902‑9.

4.	Weintraub S, Mesulam MM. Visual hemispatial inattention: stimulus parameters and exploratory strategies. Journal of Neurology, Neurosurgery & Psychiatry. 1988;51(12):1481‑8.

5.	Laurent-Vannier A, Chevignard M, Pradat-Diehl P, Abada G, Agostini MD. Assessment of unilateral spatial neglect in children using the Teddy Bear Cancellation Test. Developmental Medicine and Child Neurology. 2006;48(2):120‑5.

6.	Rinaldi L, Di Luca S, Henik A, Girelli L. Reading direction shifts visuospatial attention: An Interactive Account of attentional biases. Acta psychologica. 2014;151:98‑105.

7.	McCourt ME, Jewell G. Visuospatial attention in line bisection: stimulusmodulation of pseudoneglect. Neuropsychologia. 1999;37(7):843‑55.

8.	Bailey MJ, Riddoch MJ, Crome P. Test–retest stability of three tests for unilateral visual neglect in patients with stroke: Star Cancellation, Line Bisection, and the Baking Tray Task. Neuropsychological Rehabilitation. 2004;14(4):403‑19.

9.	Hatta T, Yoshizaki K, Ito Y, Mase M, Kabasawa H. Reliability and validity of the digit cancellation test, a brief screen of attention. Psychologia. 2012;55(4):246‑56.

10.	Roy LB, Sparing R, Fink GR, Hesse MD. Modulation of attention functions by anodal tDCS on right PPC. Neuropsychologia. 2015;74:96‑107.

11.	Loftus AM, Nicholls ME. Testing the activation–orientation account of spatial attentional asymmetries using transcranial direct current stimulation. Neuropsychologia. 2012;50(11):2573‑6.

12.	Giglia G, Mattaliano P, Puma A, Rizzo S, Fierro B, Brighina F. Neglect-like effects induced by tDCS modulation of posterior parietal cortices in healthy subjects. Brain Stimul. 2011;4(4):294‑9.

13.	Glass GV, Peckham PD, Sanders JR. Consequences of Failure to Meet Assumptions Underlying the Fixed Effects Analyses of Variance and Covariance. Review of Educational Research. sept 1972;42(3):237‑88.

14.	Lumley T, Diehr P, Emerson S, Chen L. The Importance of the Normality Assumption in Large Public Health Data Sets. Annu Rev Public Health. 2002;23(1):151‑69.

15.	Benwell CS, Learmonth G, Miniussi C, Harvey M, Thut G. Non-linear effects of transcranial direct current stimulation as a function of individual baseline performance: Evidence from biparietal tDCS influence on lateralized attention bias. Cortex. 2015;69:152‑65.

16.	Learmonth G, Felisatti F, Siriwardena N, Checketts M, Benwell CS, Märker G, et al. No interaction between tDCS current strength and baseline performance: A conceptual replication. Front Neurosci. 2017;11:664.
