# Supplementary material for: Modulation of initial leftward bias in visual search by parietal tDCS
Source: PLoS One. 2024 Dec 31;19(12):e0315715. doi: 10.1371/journal.pone.0315715 (PMC11687727; doi:10.1371/journal.pone.0315715)
Supplement: Attachment — Submitted filename: GEERS_Response_to_Reviewers_Round2.docx [file pone.0315715.s005.docx]

We thank both reviewers for their positive feedback and for recognizing the improvements we have made. We genuinely appreciated their thoughtful suggestions and carefully considered them to further enhance the manuscript. Their input has been invaluable in refining our work.

Reviewer #1: I appreciate the changes that the authors have made. The description of what the bias entails is now more accurate, and analyzing the first target position seems appropriate. I have some suggestions that the authors may want to consider.

It would be transparent to mention in the end of the introduction (and maybe also the methods and results) that the CoC was the primary endpoint, and later on secondary analyses were conducted on the starting position and total percentage of cancelled targets. Also reflect on this in the discussion section. The evidence is still valuable, but since the analysis differed from the planned analyses, the results (especially the marginal effect on the total percentage of cancelled targets in just one task) should be interpreted with caution.

We appreciate the importance of transparency and have clarified in both the Introduction (page 6, lines 123-128), Methods (page 11, lines 253-255) and Discussion (page 23, lines 501-503) that analyses on the starting position were secondary analyses. However, we would like to emphasize that analyses on the percentage of cancelled targets were planned from the start and were included in the first version of the manuscript. In the initial version, we observed a significant tDCS × hemisphere × task interaction on overall performance. Following a reviewer’s suggestion to address normality concerns, we applied a mixed-model analysis instead of an ANOVA, which resulted in a slightly different outcome—a significant tDCS × hemisphere interaction, with a marginal difference between active and sham tDCS in the right hemisphere group. Importantly, we have carefully discussed these results, particularly considering the marginal significance of the post-hoc test, to ensure an appropriately cautious interpretation (pages 22-23).

Related to the previous point, the authors stress the ‘marginally significant’ finding in the effect on the percentage of targets, whereas the non-significant findings are discussed less. I think the authors should be more cautious in the interpretation of this finding.

We agree and have revised the manuscript to adopt a more cautious tone when interpreting the marginally significant finding on the percentage of cancelled targets (page 22-23).

The authors use the x-position in pixels, which is fine for the analysis, but it would be insightful to add in the results section how much the x-position shifted in terms of visual degrees or cm. Also provide the size of the laptop screen in cm.

Thank you for pointing this out. We have added the information on the shift in x-position in terms of centimeters in the Results section (pages 13-14). Additionally, we have included the size of the tablet screen in in the Methods section to provide the necessary context for interpreting these results (page 7, lines 173-174).

Reviewer #2: The authors have addressed all the reviewer's comments. I appreciate the authors’ effort in addressing the concerns previously raised, which have significantly improved the manuscript quality making it suitable for publication. I still have few minor concerns to be addressed. Please comment on the lack of a significant visual imagery effect, which may be attributable to the fact that the Cloud task is always administered after the visual search tasks. Furthermore, no mention is made regarding the visual imagery findings in the abstract. Please consider to integrate this.

We have added a discussion on the lack of a significant of the visual imagery effect and its potential relation to task order in the Discussion section (page 24, lines 531-541). Additionally, we have integrated the visual imagery findings into the Abstract (page 2, lines 40-44).
